# Supplementary material for: Corneal Cross-Linking for Paediatric Keratoconus: A Systematic Review and Meta-Analysis
Source: J Clin Med. 2021 Jun 15;10(12):2626. doi: 10.3390/jcm10122626 (PMC8232120; doi:10.3390/jcm10122626)
Supplement: Supplementary file 1 [file jcm-10-02626-s001.zip › jcm-1204514-supplementary.pdf]

Supplementary Materials S1. Search strategy for PACK-CXL in MEDLINE.

1. Cross-link*.mp
2. Crosslink*.mp
3. CXL.mp
4. KXL.mp
5. Cross-Linking Reagents/
6. Riboflavin*.mp
7. Vitamin B.mp
8. Photosensiti*.mp
9. Paediatric.mp
10. Keratoconus*.mp
11. Corneal ectasia*.mp
12. Adolescence.mp
13. Kids.mp
14. 1 or 2 or 3 or 4 or 5 or 6 or 7 or 8
15. 9 or 10 or 11 or 12 or 13
16. 14 and 15
17. Limit 16 to humans

Supplementary Materials S2. Risk of Bias Table in Systematic Review.

First author	Year	Random sampling	Clear inclusion criteria	Confounding addressed	Objective outcome criteria	Group description	Follow up sufficient	Drop out handling	Outcome measuring	Statistics
Caporossi	2011									
Buzzonetti	2012									
Arora	2012									
Vinciguerra	2012									
Salman	2013									
Chatzis	2013									
Hashemi	2013									
Magli	2013									
Viswanathan	2014									
Kumar Kodavoor	2014									
Soeters	2014									
Ozgurhan	2014									
Shetty	2014									
Buzzonetti	2015									
Peyman	2015									
Godefrooij	2016									

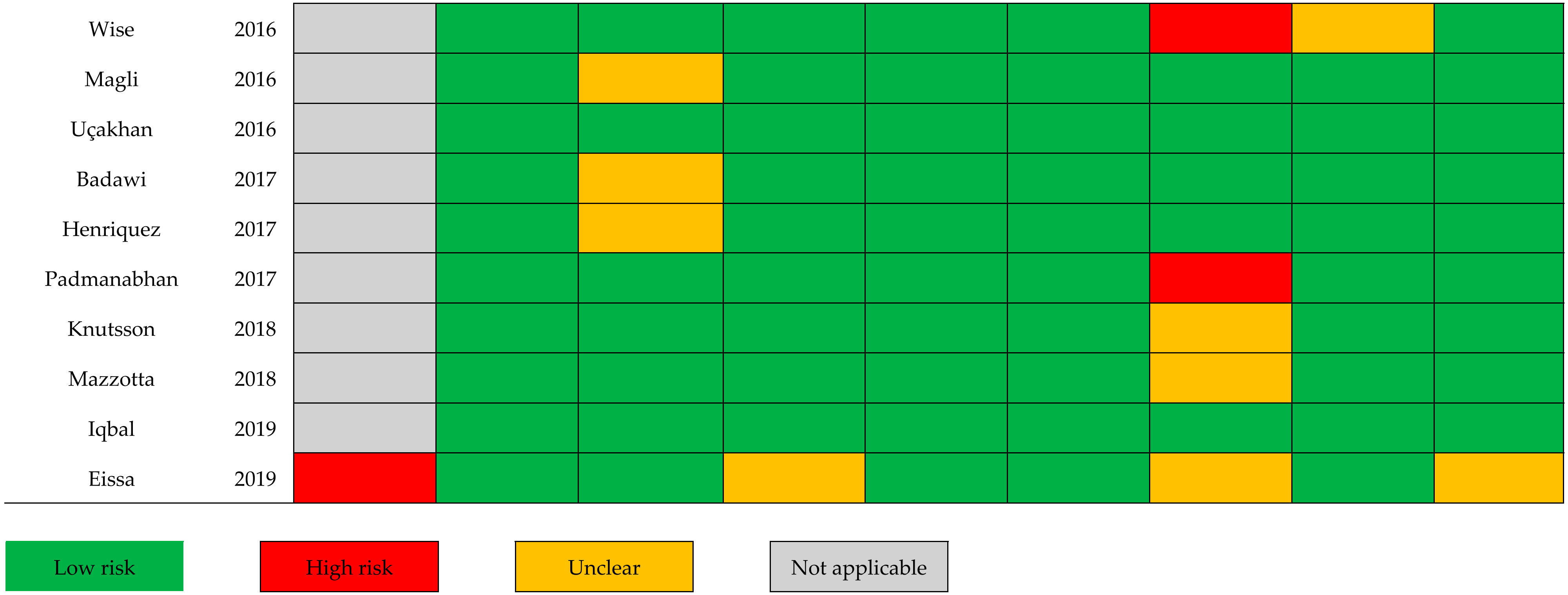
Wise 2016
Magli 2016
Uçakhan 2016
Badawi 2017
Henriquez 2017
Padmanabhan 2017
Knutsson 2018
Mazzotta 2018
Iqbal 2019
Eissa 2019
Low risk
High risk
Unclear
Not applicable
